# Applications and Prospects of CRISPR/Cas9 Technology in the Breeding of Major Tropical Crops

**DOI:** 10.3390/plants13233388

**Published:** 2024-12-02

**Authors:** Lixia Zhou, Xianhai Zeng, Yaodong Yang, Rui Li, Zhihao Zhao

**Affiliations:** 1 National Key Laboratory for Tropical Crop Breeding, Chinese Academy of Tropical Agricultural Sciences, Haikou 571101, China; lxzhou@catas.cn (L.Z.); zxh200888@126.com (X.Z.); yyang@catas.cn (Y.Y.); lirui@catas.cn (R.L.); 2 Coconut Research Institute, Chinese Academy of Tropical Agricultural Sciences, Wenchang 571339, China; 3 Chinese Academy of Tropical Agricultural Sciences, Haikou 571101, China

**Keywords:** CRISPR-Cas9, tropical crops, delivery pattern, mutation detection, application progress

## Abstract

China is a major producer of tropical crops globally, boasting rich varieties and diverse functions. Tropical crops account for two-thirds of the plant species in this country. Many crops and their products, such as oil palm, rubber, banana, sugarcane, cassava, and papaya are well known to people. Most of these products are irreplaceable and possess special functions. They not only supply important raw materials for people’s daily life and for industrial and agricultural production but also contribute to the economic growth in the tropical and subtropical regions of China. However, the modern molecular breeding of these crops is severely hampered by their biological characteristics and genetic complexity. Issues such as polyploidy, heterozygosity, vegetative propagation, long juvenile periods, and large plant sizes result in time consuming, low efficiency, and slow progress in conventional breeding of the major tropical crops. The development of genome-editing technologies has brought a new way in tropical crops breeding. As an emerging gene-editing technology, the CRISPR-Cas9 system has been widely used in plants, adopted for its higher targeting efficiency, versatility, and ease of usage. This approach has been applied in oil palm, rubber, banana, sugarcane, cassava, and papaya. This review summarized the delivery patterns, mutation detection, and application of the CRISPR-Cas9 system in tropical crop breeding, discussed the existing problems, and addressed prospects for future applications in this field, providing references to relevant studies.

## 1. Introduction

**Figure 1:**
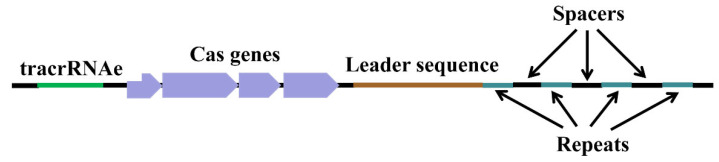
The basic structure of CRISPR/Cas.

The CRISPR/Cas system (clustered regularly interspaced short palindromic repeats/CRISPR-associated protein) has become one of the most popular and advanced tools for genome editing. It originated as early as 1987. When Ishino et al. was studying the sequence and function of the *iap* gene in *Escherichia coli*, they discovered a repetitive sequence, which was composed of 29 bp repeats and connected together by 32–33 bp non-repetitive spacer segments [1]. Subsequently, a large number of repetitive sequences with similar structures were found in the genomes of bacteria and archaea. In 2002, it was named clustered regularly interspaced short palindromic repeats (CRISPR) [2]. CRISPR is a special family of DNA repeat sequences. It mainly consists of tracrRNA, Cas gene family proteins and three sequence (leader sequence, repeat sequence, and spacer sequence) elements (Figure 1). This sequence is a defense system of prokaryotes in nature, which resists the invasion of phages, etc., by activating the adaptive immune response [3,4,5]. Through the two component system of Cas proteins and guide RNA (sgRNA and single RNA) contained in the CRISPR sequence, CRISPR/Cas can perform genetic operations such as targeted insertion, deletion, and replacement of genes in eukaryotes [6,7,8,9]. The development and application of the CRISPR/Cas system as a gene-editing tool for eukaryotic cells has completely transformed the field of genome engineering [10,11,12,13]. In 2013, the CRISPR/Cas9 system was successively applied to *Arabidopsis* [14], tobacco [15], rice, and wheat [16], showing the broad application prospects of the CRISPR/Cas9 technology in plants. Since then, the continuous improvement of the CRISPR/Cas9 system has made it a low-cost, highly efficient, and precise genetic operation tool, and it has also made plant genome editing easier and more widely applicable. More and more crops and other plant species have carried out gene function verification and trait improvement through genome editing [17,18,19,20,21]. Therefore, the CRISPR/Cas9 technology may change the prospects of traditional breeding by introducing target traits more accurately in a shorter time. More importantly, compared with transgenic breeding technology, CRISPR/Cas9 does not transfer exogenous genes into the target genome during knock-out genome editing, thereby paving the way for reducing biosafety problems [22]. 

Gene editing refers to the technology that realizes base insertion, deletion, and substitution at specific sites of the target genes through specific methods [23]. ZFN (zinc finger nuclease), TALEN (transcription activator-like effector nuclease), and the CRISPR/Cas system can all achieve gene editing [24]. Compared with ZFN and TALEN, the CRISPR/Cas system has the advantages of a simple target sequence design, a simple backbone vector construction process, and low cost [25,26]. It is the most clearly identified, most popular, and most widely used system in biological genome editing. This system consists of two components: one is an RNA-guided DNA endonuclease called Cas9, and the other is sgRNA. The combination of Cas9 and sgRNA can target the genomic sequence complementary to the sgRNA and catalyze double-stranded breaks (DSB) in the DNA backbone. Then, double-stranded breaks are mainly repaired through the error-prone non-homologous end-joining (NHEJ) pathway or the error-free homology-directed repair (HDR) [27]. Non-homologous end-joining is prone to cause single-base insertion, deletion, or substitution. Under natural conditions, the probability of homologous recombination in eukaryotic cells is very low, while the probability of non-homologous end-joining is higher. Therefore, gene modification and genome editing can be achieved through double-stranded breaks and subsequent DNA repair [28]. However, when considering the possibility of avoiding the formation of non-intact insertions, guide RNAs (e.g., CRISPR), which includes testing the developed tool not only for its editing efficiency but also for its ability to induce off-target insertions. 

Nevertheless, the biggest weakness of Cas9-based gene editing is the formation of off-target double-stranded breaks in the genome, which may generate mutations and chromosomal abnormalities, such as translocations and inversions. Currently, several methods have been developed to detect these off-target effects, such as high-throughput sequencing-based techniques. Moreover, researchers are constantly exploring new Cas9 variants or alternative gene-editing systems to reduce the off-target effects. Most pathogenic mutations and agronomically important genetic variations are SNPs (single nucleotide polymorphisms), and more precise genome-editing tools are required to correct these sequences [29]. To address the issues mentioned above, Cas9-based base editors (BEs) and prime editors (PEs) have been developed. Base editing and prime editing are two precise genome-editing tools that can perform editing at target sites without the need for double-stranded break formation or a donor DNA template [30,31]. Base editing is a fusion protein of a catalytically inactive Cas9 domain and a deaminase domain. Guided by sgRNA, base editing targets the target base sequence, thereby achieving the purpose of modifying the target gene. The base-editing tools successively developed by researchers mainly include the following: CBE (cytosin base editor) that can achieve C:G to T:A conversion, ABE (glycosylase base editor) that can achieve A:T to G:C conversion, CGBE (C to G base editor) that can achieve C:G to G:C conversion, and a dual deaminase-mediated base editor made by fusing CGBE with ABE (AGBE) that can simultaneously generate four types of base conversions (C to G, C to T, C to A, and A to G) as well as InDels [32,33,34]. Compared with base editing, prime editing can perform all possible base conversions, including small InDels and their combinations, at the target site. Currently, plant-based base-editing and prime-editing systems have been tested in several plants such as rice [35,36], wheat [37], corn, and tomato [38,39]. Tropical crops including oil palm, rubber, banana, sugarcane, cassava, and papaya grown in tropical regions supply important raw materials for our daily life, industry, and agriculture. However, modern breeding of these crops is severely hampered by their biological characteristics and genetic complexity. Although the CRISPR/Cas9 technology has been reported in some studies on tropical crops, the relevant research is still in its infancy. Compared with model crops like *Arabidopsis*, rice, and wheat, the research and application level of CRISPR/Cas9 in tropical crops lags far behind. We present the research trends of the CRISPR/Cas9 system and review its application in tropical crops, aiming to offer references for the genome-editing breeding of tropical crops.

## 2. Transformation and Mutation Detection Methods of the CRISPR/Cas9 in Plants

### 2.1. The Modes of Transformation

For plants, common genetic transformation methods include *Agrobacterium*-mediated genetic transformation [40], biolistic-mediated transformation (gene gun) [41], and protoplast transformation [42,43]. Currently, Agrobacterium-mediated genetic transformation is often used for gene editing in oil crops. In this method, the T-DNA region with Cas9 protein and sgRNA expression cassettes is randomly integrated into the plant genome. Therefore, the Cas9 protein in the T-DNA region has persistent activity, and edited plant progeny can obtain edited individuals without T-DNA [44]. Sometimes, the vector backbone can also be inserted into the plant genome, and the T-DNA is prone to insert multiple copies during integration. Thus, it is more difficult to obtain truly non-transgenic individuals, and because of the random insertion location, it may affect the expression of other genes [45]. The gene gun method uses power to penetrate the cell walls and cell membranes of plants to introduce exogenous CRISPR/Cas9 vectors; once the vectors finally enter the plant cells, they are randomly integrated into the plant genome [46]. The gene gun method has a simple vector construction process and no host genotype limitation, but its parameter settings are complex, and it is easy to insert multiple copies, resulting in gene silencing and genetic instability in progeny [47]. The protoplast transformation method directly introduces plasmid vectors with gene editing into cells [43]. Compared with other transformation methods, the protoplast transformation method has a higher transformation efficiency. However, for most plants, compared with receptors such as callus, meristem, and leaves, the protoplast preparation process is more complicated. In order to increase the targeting specificity of the vector, the time that the active substances remain active in the cells has to be reduced; To prevent the introduction of exogenous DNA fragments, researchers pre-assemble purified Cas9 protein and artificially synthesized sgRNA into a ribonucleoprotein complex (RNP) in vitro, and then transfect this complex into plant tissues to achieve gene editing [48]. In addition, the introduction methods of CRISPR/Cas9 also include the pollen-tube pathway method [49], electroporation method [50], microinjection method, etc. [51].

### 2.2. Mutation Detection Methods

Regarding the detection of single-plant mutations edited by CRISPR/Cas9, many methods have been developed so far. For example, the commonly used PCR restriction enzyme (PCR-RE) detection [52], T7 endonuclease I (T7EI) detection [53], surveyor enzyme detection, genotyping assays based on polyacrylamide gel electrophoresis (PAGE) [54], assays based on high-resolution melting (HRM) [55], mutant discrimination based on Sanger sequencing, and high-throughput sequencing [56].

The principle of PCR-RE is to select a target sequence containing a restriction enzyme site when choosing the target sequence. If a mutation occurs, the amplified sequence containing the target site cannot be cleaved by the endonuclease during detection [52]. Therefore, although this method has high sensitivity, its applicable range is not wide. T7EI and surveyor enzymes can both cleave genomes with DNA hybridization mismatches, so there are no restrictions on the selection of target sequences, but their sensitivity is not as high as that of PCR-RE [53]. The principle of PAGE-based genotyping detection is that the conformation of single-stranded DNA changes with the change in bases and DNAs with different conformations have different migration rates in denaturing polyacrylamide gels [54]. Therefore, the PAGE method is also used for mutation detection of the progeny edited by the CRISPR/Cas9 system. This method is labor-intensive, has a large workload, and takes a long time, but it has high sensitivity. HRM is based on the fact that different base sequences have different melting temperatures, but this method requires an expensive device for analysis and has relatively low sensitivity [57]. Based on the same principle, Hua et al. developed an annealing at critical temperature PCR (ACT-PCR) based on conventional PCR to detect whether mutations occur in the edited progeny. ACT-PCR only requires agarose gel electrophoresis after a single PCR step, so the steps are simple, and the cost is low, and the sensitivity is high [58].

Researchers have also developed other mutation detection methods for cases with a low mutation frequency and a large detection population. For example, the in vitro assembled RNP is used to cleave the PCR products of edited wheat (*Triticum aestivum*) or rice (*Oryza sativa*). The results show that this method is more sensitive than Sanger sequencing and more applicable than the PCR-RE method, without the restriction of restriction enzyme site selection [59]. Li et al. reported a strategy for generating and screening amino acid point mutations using the CRISPR/Cas9 gene-editing system. This strategy is based on utilizing codon degeneracy to design donor DNA, which enables the design of specific primers to facilitate mutant screening through PCR. The assembled ribonucleoprotein (RNP) complex was electroporated into wild-type cells along with double-stranded DNA donors and antibiotic resistance markers, followed by PCR screening [60]. All of the above methods can only detect whether mutations occur in the target sequence, and the specific mutation types need to be obtained from sequencing results. High-throughput sequencing based on software analysis has high accuracy and sensitivity in mutation detection but is expensive. Similarly, Sanger sequencing is also relatively expensive, especially when there are a large number of positive seedlings obtained. Therefore, the best approach is to determine in advance whether the target sequence has been edited before sequencing.

## 3. The Application of CRISPR/Cas9 Technology in Major Tropical Crops

The CRISPR/Cas9 system is theoretically applicable to all species. There are already mature application systems in model plants, and it has been preliminarily applied in major tropical crops such as oil palm, rubber, bananas, sugarcane, cassava, and papayas. The application progress is elaborated in Table 1.

### 3.1. Oil Palm

The oil palm (*Elaeis guineensis* Jacq.), the king of oils in the world, is an important tropical woody oil-bearing crop and the main raw material crop for palm (kernel) oil. The global demand for palm oil is increasing year by year. By 2050, the demand for palm oil will increase to 250 million tons, exceeding the total output of oils and fats in the world [86]. Breeding high-yielding oil palms with improved traits is a prerequisite for meeting the growing consumer demand for palm oil. The oil palm belongs to monocotyledonous cross-pollinated plants in the Palmaceae family, and its progeny has large trait variations. It is difficult to meet commercial requirements with conventional cross-breeding strategies. The bi-breeding technique has become an ideal choice for oil palm breeding. Researchers have optimized the transgenic technology of oil palm and obtained transgenic seedlings or transgenic calli [87]. 

In order to establish an efficient CRISPR/Cas9 system in oil palm, researchers designed five gRNAs targeting phytoene desaturase (*EgPDS*) gene. Using the rapid electroporation-mediated protoplast transient expression system, they detected the function of the codon-optimized SpCas9 and the effectiveness of the five gRNAs to identify efficient gRNAs [61]. The results showed that the electroporation transfection efficiency of oil palm mesophyll protoplasts was from 17% to 26%, and the cleavage frequencies detected in the protoplasts transformed with Cas9/*EgPDS4* and Cas9/*EgPDS5* were 6.49% and 25.49%, respectively. Yeap et al. further used biolistic bombardment to transform immature zygotic embryos of oil palm to edit the target sites of the *EgPDS* and brassinosteroid receptor protein gene (*EgBRI1*, *brassinosteroid-insensitive 1*), achieving a relatively high editing efficiency (from 42.86% to 100%). In the oil palm seedlings edited using *EgPDS*, a chimeric albino phenotype was observed, while in the oil palm seedlings edited by *EgBRI1*, poor growth and development and leaf tip necrosis phenotypes were exhibited [61]. Bahariah et al. first used the CRISPR/Cas9 technology to target genes related to high oleic acid in oil palm. Based on the sequences of *EgFAD2* and *EgPAT*, a total of four single-guide RNAs (sgRNAs) were designed in silico. With the powerful plant CRISPR/Cas9 vector technology, multiple sgRNA expression cassettes were effectively constructed into a single binary CRISPR/Cas9 vector to edit the *EgFAD2* and *EgPAT* genes. Then, each constructed transformation vector was delivered into oil palm embryogenic calli using biolistic, Agrobacterium-mediated, and PEG-mediated protoplast transformation methods. The sequence analysis of PCR products from 15 samples confirmed that mutations were introduced at four target sites of *EgFAD2* and *EgPAT* genes. Single-knockout and double-knockout mutants were generated for both genes, with large and small deletions within the targeted regions. The mutations found at the *EgFAD2* and *EgPAT* target sites indicated that the Cas9/sgRNA genome-editing system effectively knocked out these two genes in oil palm [62]. These results achieved effective gene editing in oil palm by optimizing the selection of highly efficient gRNAs and DNA delivery methods, providing a new approach for the genetic improvement of oil palm.

### 3.2. Rubber Tree

The rubber tree (*Hevea brasiliensis* Muell. Arg.) is currently the main source of natural rubber. As a cross-pollinated tree species with a long juvenile period (from 6 to 7 years), the efficiency of genetic improvement of rubber trees through conventional breeding methods is very low, making it difficult to meet economic demand [88]. Therefore, there is an urgent need for more effective molecular methods to accelerate the improvement of rubber tree traits. 

The complete sequencing of the whole genome sequence of rubber trees and the successful establishment of the method for regenerating rubber tree protoplasts have laid the foundation for gene editing in rubber trees using the CRISPR/Cas9 technology. Liu used the CRISPR/Cas9 technology to target *AtMlo2* transgenic plants of *Arabidopsis* dermatophyte powdery mildew mutant pad4, laying the foundation for the further cultivation of rubber tree powdery mildew-resistant strains [89]. Fan et al. first attempted to develop a DNA-free genome-editing system using CRISPR/Cas9 ribonucleoprotein in rubber tree protoplasts. The team designed sgRNAs for genes (*FT* and *TFL1*) that can accelerate and delay the flowering period of rubber trees, and then used the PEG-mediated method to assemble Cas9 sgRNA into ribonucleoprotein (RNP), which was then introduced into leaf mesophyll cells to prepare the protoplasts. They were the first to establish a CRISPR/Cas9 system for rubber trees internationally, providing a reference for cultivating gene-edited rubber strains without exogenous DNA contamination [63]. In 2021, the team further utilized five endogenous U6 promoters from rubber trees to drive the transcription of sgRNA. Transient editing was performed in Brazilian rubber tree protoplasts, and it was found that HbU6-2 and pHbU6-4 had the highest editing efficiency. The shortest promoter HbU6-2 was selected as the promoter for subsequent experiments [64]. Subsequently, Dai et al. used ten sgRNAs targeting five flowering-related genes (*FT* and *TFLl*) to perform genome-editing analysis on rubber tree protoplasts, and they discovered three mutation patterns, namely deletion, insertion, and base substitution. Among them, short deletion was the most prominent, and a stable transformation editing vector targeting *HbPDS* gene was constructed. It was transformed into rubber tree callus tissue using a CRISPR/Cas9-mediated gene-editing-induced homozygous mutant callus tissue and the expected albino phenotype was obtained [65]. Yang et al. transformed a CRISPR vector targeting the Phytoene desaturase gene (*HbPDS*) into embryos and obtained genome-edited rubber trees with expected phenotypes. This study has made significant progress in transgenic and gene editing of rubber trees [66].

### 3.3. Banana

Banana (*Musa* spp.) is an economically important crop that serves as both a fruit and a food staple around the world. It is also the fruit with the largest global fresh-fruit consumption and trade volume, playing an important role in promoting poverty alleviation and rural revitalization among farmers in tropical and subtropical areas in China. However, banana production is severely threatened by biotic and abiotic stresses such as fusarium wilt, drought, and chilling injury. The most important banana cultivars worldwide are triploid, highly sterile, and have a narrow genetic base. These characteristics seriously hinder the application of traditional cross-breeding techniques to cultivate excellent new banana varieties. Moreover, mutagenesis breeding and mutant selection for new varieties have problems such as long cycles and great blindness. Therefore, banana bio-breeding is considered an ideal approach for cultivating new varieties with high quality, stress resistance, and disease resistance traits.

Researchers have successively established the CRISPR/Cas9 genome-editing technology system in bananas. Hu et al. used the CRISPR/Cas9 gene-editing technology to edit the phytoene desaturase gene (*MaPDS*) in Brazilian bananas (AAA), obtaining completely albino and variegated-leaf heterozygous banana mutant lines with an editing efficiency of 55% [67]. Kaur et al. designed sgRNAs targeting two *PDS* genes (*RAS-PDS1* and *RAS-PDS2*) in Rasthali (AAB) to guide Cas9 to cut the target genes, and the mutation efficiency reached 59% [68]. Naim et al. designed two sgRNAs targeting the first exon of the *PDS* gene in Williams (AAAA) to guide Cas9 to cut the target genes, and the editing efficiency reached 100% [90]. Ntui et al. compared the *PDS* genes between the *Musa accuminata* (AA) and *Musa balbisiana* (BB) reference genomes, designed two sgRNAs targeting the *PDS* gene in the most conserved region to construct a CRISPR/Cas9 vector, and transformed it into the embryogenic suspension cells of Sukali Ndiizi (banana) (AAB) and Oonja Manjaya (plantain) (AAB). After sequencing the target sites of 18 edited lines, highly efficient mutations of 100% and a large-fragment deletion of 723 bp were observed in both varieties [69]. Regarding the optimization and improvement of the banana gene-editing system, Wu et al. compared the editing efficiencies of CRISPR/Cas9, CRISPR/Cas12a, and RNP-CRISPR-Cas9 for the *PDS* gene in banana protoplasts and found that the mutagenesis efficiency mediated by CRISPR/Cas9 was higher than the other two systems [91]. Zhang et al. optimized Cas9 by codon optimization and used the banana endogenous U6 promoter to optimize the Cas9 genome-editing backbone vector, increasing the editing efficiency by four times [70]. Hu et al. used the CRISPR/Cas9 genome-editing technology to integrate the genome-editing elements and gene-clearing elements on the same vector, achieving the deletion of the introduced functional gene components after genome editing occurred at the target site, thereby establishing an efficient genome-editing technology system without transgenic residues in bananas, laying the foundation for the industrial application of banana genome-editing technology [92].

With the successful establishment of the CRISPR/Cas9-editing technology system in bananas, the CRISPR/Cas9-mediated genome-editing technology has been applied in bananas in aspects such as enhancing disease resistance, improving fruit quality, extending shelf-life, and optimizing plant-type structure. Banana streak virus (BSV) is a double-stranded rod-shaped DNA virus integrated into the B genome of bananas, known as endogenous BSV (eBSV). It seriously affects the production of African plantains (AAB) and also restricts the use of the diploid ancestral species *Musa balbisiana* carrying the B genome or its derivatives as parents. Tripathi et al. used the CRISPR/Cas9 technology to edit the endogenous banana streak virus, making it inactive and unable to produce infectious virus particles, paving the way for banana production, cross-breeding, and the application of the B genome in the genetic improvement of plantains, providing an effective strategy for inactivating other endogenous viruses [93]. Tripathi et al. used CRISPR/Cas9 to edit the *MusaDMR6* gene and obtained bananas resistant to Xanthomonas wilt [71,72]. Researchers have also attempted to cultivate new varieties resistant to *Fusarium oxysporum* Schlecht f.sp. *cubense* (E.F.Sm.) Snyd.et Hans (Race 4) through the CRISPR technology [94]. The carotene content in the pulp of most Cavendish banana varieties is relatively low. Kaur et al. used the CRISPR/Cas9 technology to edit the fifth exon of the banana *LCY_Ɛ_* gene to create β-carotene-rich banana varieties, obtaining edited lines with a six-fold increase in β-carotene content in the fruits [95]. Research has found that the higher expression of *CCD4* (*RAS-CCD4*) in Rasthali (AAB) is negatively correlated with the β-carotene accumulation in the pulp. Awasthi used the CRISPR/Cas9 technology to edit the *RAS-CCD4* gene in Rasthali and found that the β-carotene accumulation in the roots of the mutant lines increased more than that in the leaves [73]. Editing the *MaGA200x2* [74], *Achn379131* [75], and *MaAC01* [76] genes in bananas, respectively, also resulted in mutant lines with semi-dwarfing and extended shelf-life of banana fruits.

### 3.4. Sugarcane

Sugarcane (*Saccharum* spp.) is an industrial raw material crop that produces 80% of the world’s sugar and 26% of bioethanol [96]. The genome of sugarcane is the most complex among all domesticated agricultural species (2n = 100 to 130), and its complex and highly polyploid genome hinders the application of traditional hybrid breeding techniques in sugarcane variety improvement. Genome editing will usher in a new era of precision breeding for sugarcane [97,98].

Arencibia et al. established a basic Agrobacterium-mediated sugarcane genetic transformation system in 1998, which provided a reference for subsequent research [99]. Enríquez-Obregón et al. used Agrobacterium to infect sugarcane callus and obtained transgenic sugarcane plants resistant to herbicides [100]. Matusuoka et al. carried out genetic transformation using callus and cell suspension cultures of sugarcane varieties. Their binary vector pMLH7133-GUS contains the neomycin phosphotransferase *nptII* gene, the hygromycin phosphotransferase *hpt* gene, and the *uidA* gene, and the Agrobacterium tumefaciens strains were *EHA101* and *LBA4404* [77]. Zhao et al. detected the transformation effects of the Cre/lox and CRISPR/Cas9 systems using the method of silencing–restoring GUS activity and compared their specific recombination efficiencies at the same time. The results showed that the recombination efficiency of the optimized Cre/lox system was higher than that of the CRISPR/Cas9 system, and the recombination efficiency of the Cre/lox system was higher than that of the CRISPR/Cas9 system [101]. It has been verified that the mutation effect of the CRISPR/Cas9 system is in full accordance with homologous recombination, which can achieve targeted and precise mutations and provide a reliable basis for the experimental design of targeted gene editing in sugarcane. Magnesium chelatase (MgCh) is a key enzyme in the chlorophyll biosynthesis pathway. Different from the dwarf and albino phenotypes of the PDS-knockout gene, MgCh mutants show a light-green-to-yellow leaf phenotype and have a growth rate similar to that of the wild type. Eid et al. designed two sgRNAs targeting the MgCh gene, constructed a vector with Cas9 whose codons were optimized and driven by the CaMV35S promoter, and transformed the callus of sugarcane variety CP88.1762 via the gene gun method. They also compared the influence of culturing the callus at 28 °C or culturing it at 37 °C for 48 h after transformation for 4 days and then transferring it to 28 °C on the mutation frequency. It was found that heat treatment doubled the editing efficiency and greatly promoted the co-mutation of multiple alleles. The phenotypes of the descendants of co-mutation are the most obvious, with severe chloroplast depletion [78]. Meanwhile, Oz et al. used the CRISPR/Cas9 technology to edit two codons (W574L and S653I) related to herbicide resistance in acetolactate synthase (ALS) of sugarcane. The evaluation of 146 independently transformed plants from five independent experiments showed that, except for 25 or 18 strains of W574L or S653I, amino acid substitutions targeting both W574L and S653I simultaneously occurred in the acetolactate synthase (ALS) of 11 strains. This work can convert inferior alleles into superior alleles through targeted nucleotide substitution [79].

### 3.5. Cassava

Cassava (*Manihot esculenta* Crantz) is an important staple crop widely planted in tropical and subtropical regions around the world, and it is also a major industrial raw material [102]. Effective gene editing in this important staple crop will provide new opportunities for solving the limitations of biotic and abiotic stresses in cassava production and post-harvest utilization. The establishment of the cassava genetic transformation system and the completion of genome sequencing have enabled the potential of CRISPR-based genome-editing technology in basic and applied cassava research to be realized, thereby improving important economic traits of cassava [103]. Odipio et al. first reported the CRISPR/Cas9-mediated *MePDS*-targeted gene-editing research in two cassava varieties (TMS60444 and TME204). Fifty-eight and twenty-five independent transgenic lines were obtained in the two cassava varieties, respectively, and more than 90% of the regenerated plants of the cassava variety TME204 had an albino phenotype [104]. This opened the prelude to the application of the CRISPR/Cas9 technology in cassava.

Researchers have successively applied the CRISPR/Cas9 technology to studies such as enhancing the disease resistance of cassava [105], improving starch quality [106], and developing cyanide-free cassava varieties [107]. Cassava brown streak disease (CBSD) and cassava mosaic disease (CMD) are two major devastating viral diseases in cassava. Gomez et al. carried out targeted mutagenesis on two eukaryotic translation initiation factor 4E (eIF4E) protein subtypes, novel cap-binding protein-1 (nCBP-1) and nCBP-2 in cassava variety TMS60444, reducing the levels of cassava brown streak-related disease symptoms and the accumulation of cassava brown streak virus in storage roots [108]. Although the resistance to cassava brown streak disease was enhanced, complete resistance was not obtained. Mehta used the CRISPR/Cas9 technology to develop resistance to African cassava mosaic virus, but the edited lines did not show obvious resistance. This may be related to the fact that the researcher only used a single sgRNA targeting one viral region (AC2) [109]. Ye used the CRISPR/Cas9 technology to target and edit the genomic DNA of Sri Lankan cassava mosaic virus, screened sgRNA targets for excellent disease-resistant effects and transformed them into South China 8, the main cassava variety in China [110]. Veley et al., based on the CRISPR-mediated gene-editing technology, developed a visual tool to monitor the in vivo infection process of cassava bacterial blight (CBB) by using the GFP-marked sugar efflux transporter gene (SWEET, sugars will eventually be exported transporters) MeSWEET10ct at the 3’-end of the endogenous site [80]. This was the first demonstration of CRISPR-mediated homologous recombination and gene marking in cassava in vivo. Wang enhanced the resistance of mutant lines to cassava bacterial blight by editing the promoter of the cassava MeSWEET10a gene through the CRISPR/Cas9 technology without affecting the normal growth and yield of mutant plants [81]. In terms of improving starch traits, Bull et al. reported the application of CRISPR/Cas9 in manipulating starch biosynthesis and improving starch storage quality [111]. Luo et al. achieved gene editing of starch branching enzyme (SBE) SBE2 in cassava using the double-sgRNA CRISPR/Cas9 system and obtained mutant lines with significantly increased amylose and starch resistance. Researchers have also attempted to use the CRISPR/Cas9 technology to develop cyanide-free cassava varieties [106]. Juma et al. carried out targeted mutagenesis on exon 3 of the *MeCYP79D1* gene, which belongs to the CYP79 family of P450 monooxygenase genes (CYP, cytochrome P450 enzyme), using CRISPR/Cas9. The contents of linamarin and transmuted cyanide in the leaves of *mecyp79d1*-edited lines were reduced by 7-fold [107]. Gomez et al. further used the CRISPR/Cas9 technology to edit two key enzyme genes, *CYP79D1* and *CYP79D2,* as the first step of cyanogenic glucoside biosynthesis and found that double knock-out eliminated the cyanogenic capacity of three cassava varieties (TMS60444, TME419, and TMS9I/02324), and single-gene knock-out showed that the two CYP79D genes had different contributions to cassava cyanogenesis [82].

Currently, the model variety for cassava genetic transformation is the TMS60444 variety originating from Africa [83]. This variety has a low yield and low starch content, many branches, and poor disease resistance, and it is not an ideal biological breeding receptor material. Therefore, it is of great significance to develop an efficient genetic transformation technology for excellent cassava varieties in China. Wang et al. has made new progress in cassava biological breeding technology research. An efficient genetic transformation and gene-editing system for South China 8, the main cassava variety in China, has been established. The team drove the expression of Cas9 protein by using the YAO promoter which is highly expressed in the meristem, and the editing efficiency of the MePDS gene reached 93%, and the mono-allelic homozygous mutation rate reached 45%, which is the highest homozygous mutation rate in the field of cassava gene editing so far [84]. The research team has also successfully edited target sites such as soluble starch synthase III (SS) in cassava, namely MeSSm-J and MeSSIII-2, hexose transporter genes (STP) *MeSTP7* and *MeSTP15*, vacuolar invertase (VINV) *MeVINV1*, and *WRKY* transcription factor family gene *MeWRKY12* using the CRISPR/Cas9 technology, which lays the foundation for obtaining relevant mutants and further analyzing the mechanisms of cassava tuberous root development, cassava starch synthesis, and accumulation [112].

### 3.6. Papaya

Papaya (*Carica papaya* L.) is a perennial herbaceous fruit tree of the genus Carica in the Caricaceae family. It is widely planted in tropical and subtropical countries and is popular because of its rich nutrition and medicinal value [113]. However, the development of the papaya industry is severely restricted by papaya ringspot virus disease (PRSV), and the cultivation of disease-resistant varieties has become the key to the development of this industry [114]. Due to the lack of disease-resistant resources for edible papaya in China and the narrow genetic basis of cultivated species, it is difficult to cultivate disease-resistant varieties through traditional cross-breeding, while molecular breeding techniques can effectively improve the disease-resistance of varieties. Transgenic papaya resistant to papaya ringspot virus was the first transgenic fruit crop to be commercialized in Hawaii in 1998 [115]. The papaya genome sequence was sequenced [116], and it provided strong technical support for the precise molecular-based breeding of papaya using the CRISPR/Cas9 system [117].

Wang et al. constructed a tandem multi-cleavage-site expression vector of CRISPR/Cas9 for papaya leaf-curl virus using the Golden Gate method [118]. Subsequently, based on different conserved sequences of papaya ringspot mosaic virus, using the CRISPR/Cas9 technology and the Golden Gate vector construction system, they tandem-linked five sgRNAs to the same vector and achieved the suppression of papaya ringspot mosaic virus through transient expression in papaya leaves [119]. Recently, Brewer et al. designed three sgRNAs targeting the papaya *PDS* gene through Agrobacterium-mediated transformation and established an effective papaya gene-editing system based on CRISPR/Cas9. The edited transgenic plants showed a complete albino phenotype, and multiple gene edits including insertions, inversions, and deletions were detected at the three gRNA target sites, and the efficiency of InDel mutations was 81% [120]. In addition, Hoang et al. developed an in vivo hairy root system to evaluate the efficiency of papaya genome editing through rhizobia [121]. These studies have opened up new ways for papaya gene-function analysis, trait improvement, and breeding using the CRISPR/Cas9 system.

## 4. Limitations and Future Perspectives

Compared with model plants and staple crops, the application of the CRISPR/Cas9 system in gene function research and variety improvement of tropical crops is relatively lagging behind, and most are still in the initial stage of establishing gene-editing systems. An efficient genetic transformation and regeneration system is a prerequisite for transgenic and gene-editing operations. Although effective regeneration and genetic transformation systems have been established in the above mentioned tropical crops at present, there are still major problems. For example, in bananas, the reported transformation receptors include banana embryogenic cell suspension systems (ECSs), protoplasts, shoot-tip or multi-bud slices, etc., but each receptor has obvious drawbacks [70]. For example, although the ECS method can obtain transgenic lines originating from single cells, establishing and maintaining ECS requires a great deal of time and energy, and it has a long regeneration cycle (from 15 to 18 months), and the ECS induction technology is still limited to a few laboratories and a few genotypic varieties, with poor technical reproducibility [85]. Although the shoot-tip or multi-bud slices are simple and fast, the success rate is extremely low, and chimeras are very likely to be produced, resulting in the loss of target traits during the later screening process. Although protoplasts are convenient for transformation, their regeneration is very difficult [85]. Therefore, there is an urgent need for a widely applicable, efficient, and stable banana genetic transformation and regeneration scheme. As a monocotyledonous perennial woody oil crop, at present, the main receptor for oil palm genetic transformation is embryogenic callus [35]. Immature inflorescences are relatively ideal explant materials for inducing somatic embryo regeneration in oil palms, but the frequency of somatic embryo formation in oil palm callus is low, and bud regeneration is difficult [122]. The process from somatic embryogenesis to obtaining regenerated plant lines usually takes more than 2 years, with the somatic embryogenesis process being the most challenging, typically requiring 6–8 months. Therefore, further exploration is still required to construct an efficient and stable genetic transformation system suitable for oil palm [123]. In comparison, relevant research teams in China have established relatively mature genetic transformation and regeneration technologies with embryogenic callus as the receptor in tropical fruit papaya and tropical economic crops such as sugarcane, cassava, and rubber, but the genomic complexity including their heterozygosity and polyploidy has led to difficulties in applying genome editing to these species, and the editing efficiency needs to be improved [82,124].

Overall, although the whole genome sequencing work of the above mentioned tropical crops has been completed and published one after another, the analysis of gene pathways and gene functions related to important agronomic traits still needs to be further strengthened. Most tropical crops do not yet have a stable genetic transformation system or have a low transformation efficiency, which makes genome editing experiments and optimization difficult [125]. To obtain stably inheritable gene-edited materials, a large amount of work is required, and it is time consuming. The polyploid characteristics and genomic heterozygosity of tropical crops increase the difficulty of obtaining mutant phenotypes of target genes in the T0 generation of genetic transformation [126]. The existing plant sgRNA online design software databases only contain the genomic information of a few tropical crops. Collecting genomic data of more tropical crop varieties will be helpful for the application of CRISPR/Cas9.

Using the transient protoplast system for PEG-mediated CRISPR-element delivery is very useful for gRNA verification, but only a very few plant species can be regenerated from protoplasts. Micro-particle bombardment (gene gun) and Agrobacterium-mediated delivery are highly dependent on plant species, genotype, and tissue-type specificity [127]. To overcome genotype dependence, using specific morphogenetic factors to recombine somatic cells and make them start embryogenesis is a major breakthrough in the field of plant transformation, especially for monocotyledonous plants and difficult-to-transform plants. For example, the ectopic expression of maize embryo-autonomous-occurrence genes (*BBM*) and *WOX* transcription factor (*WUS2*) genes has increased the transformation efficiency of several crops, including maize, sorghum, indica rice, and sugarcane [128]. The constitutive expression of *WOX* transcription factor (*WOX5*) or GRF4-GIF1 chimeric proteins has increased the transformation efficiency of wheat, other monocotyledonous, and dicotyledonous plants [129,130]. Therefore, it is necessary to design and clone CRISPR/Cas9 vectors containing chimeric complexes of morphogenetic factors such as GRF4-GIF1 and apply them to tropical crops to produce stable transformants with higher regeneration efficiency.

Cao et al. has developed a delivery system (CDB, cut-dip-budding), which takes advantage of root–stock regeneration and uses Agrobacterium rhizogenes to transform and edit sweet potatoes, directly producing hairy roots [131]. This technology shows great potential in plant species that are difficult to regenerate anew from explants during the tissue culture process. To solve the limitations of genome editing in tropical crops, concentrating on developing non-tissue-culture-based transformation systems is also an important research area. For example, nanoparticle-assisted direct transgenic delivery or virus-mediated transgenic silencing of transgenes (VIGS), and then regenerating plants from infected tissues.

Wolabu et al. increased the mutagenesis efficiency of the *MsSGR* gene by 23–49% in alfalfa using a multi-gRNA-CRISPR/Cas9 vector and produced homozygous mutants with complete knockout of four allele copies in the T0 generation. This multi-gRNA-CRISPR/Cas9 genome-editing system provides a reliable and effective method for gene function identification [132]. Another strategy of the multi-gRNA-CRISPR/Cas9 genome-editing system is BREEDIT (a multi-gene-editing tool), which has been reported in maize for improving its quantitative traits [133]. Using this strategy to edit multiple genes related to plant growth, a library of more than 1000 genetically modified maize mutants was obtained. Recently, Gupta et al. developed a modular multi-gene prime-editing (PE) system, which can edit up to four gene loci in rice simultaneously. They mainly used Golden Gate cloning and Gateway recombination techniques to simplify the assembly of multiple pegPmA-ngRNA modules. Using this system, Gupta et al. carried out simultaneous two-gene (double), three-gene (triple), and four-gene (quadruple) editing experiments and achieved high editing efficiencies, simplifying the process of constructing multi-gene-editing PE vectors and enabling more laboratories to conduct multi-gene-editing experiments using PE [134]. The above studies can serve as good references for tropical crops with complex genomes and polyploidization.

Advances in genome editing and molecular biology research have led to the design of various genome-editing tools with wide applications. Therefore, it is crucial to select the best system for editing a given species and the purpose of editing. Recently, emerging base-editing and prime-editing technologies have the potential to expand the scope and efficiency of genome editing, and their applications in tropical crops can play a crucial role in precise and accelerated genetic improvement.

## Figures and Tables

**Table 1:** Application of CRISP/Cas 9 technology in major tropical crops.

Species	Promoter of Cas9 Nuclease	Target Gene	Conversion Material or Method	Reference
Oil palm	CaMV35S	EgPDS	protoplast	[61]
	CaMV35S	EgBRI1	protoplast	[61]
	CaMV35S	EgFAD2, EgPAT	protoplast	[62]
Rubber	CaMV35S	HbFT, HbTFL1	protoplast	[63]
	CaMV35S	HbU6-2	protoplast	[64]
	CaMV35S	HbPDS	protoplast	[65,66]
Banana	CaMV35S	MaPDS	protoplast	[67]
	CaMV35S	RAS-PDS1, RAS-PDS2	protoplast	[68]
	CaMV35S	MaPDS	Cell suspension	[69]
	CaMV35S	U6	protoplast	[70]
	CaMV35S	MusaDMR6	protoplast	[71,72]
	CaMV35S	RAS-CCD4	protoplast	[73]
	CaMV35S	MaGA200x2	protoplast	[74]
	CaMV35S	Achn379131	protoplast	[75]
	CaMV35S	MaAC01	protoplast	[76]
Sugarcane	CaMV35S	hpt, uidA	protoplast	[77]
	CaMV35S	MgCh	protoplast	[78]
	CaMV35S	W574L, S653I	protoplast	[79]
Cassava	CaMV35S	MeSWEET10ct	protoplast	[80]
	CaMV35S	MeSWEET10a	protoplast	[81]
	CaMV35S	SBE2	protoplast	[82]
	CaMV35S	MeCYP79D1	protoplast	[83]
	CaMV35S	CYP79D2	protoplast	[84]
Papaya	CaMV35S	PDSs	protoplast	[85]
